# Comparative pharmacokinetics and tissue distribution of primaquine enantiomers in mice

**DOI:** 10.1186/s12936-022-04054-4

**Published:** 2022-02-05

**Authors:** Pius S. Fasinu, Narayan D. Chaurasiya, N. P. Dhammika Nanayakkara, Yan‑Hong Wang, H. M. T. Bandara Herath, Bharathi Avula, James D. McChesney, David Jollow, Larry A. Walker, Babu L. Tekwani

**Affiliations:** 1 Department of Pharmacology & Toxicology, University of Alabama at Birmingham, Birmingham, AL 35294 USA; 2 Department of Infectious Diseases, Division of Scientific Platforms, Southern Research, Birmingham, AL 35205 USA; 3 National Center for Natural Products Research, Research Institute of Pharmaceutical Sciences, School of Pharmacy, The University of Mississippi, University, MS 38677 USA; 4 Cloaked Therapeutics, Inc., Etta, MS 38627 USA; 5 Department of Pharmacology, Medical University of South Carolina, Charleston, SC USA

## Abstract

**Background:**

Primaquine (PQ) has been used for the radical cure of relapsing *Plasmodium vivax* malaria for more than 60 years. PQ is also recommended for prophylaxis and prevention of transmission of *Plasmodium falciparum*. However, clinical utility of PQ has been limited due to toxicity in individuals with genetic deficiencies in glucose 6-phosphate dehydrogenase (G6PD). PQ is currently approved for clinical use as a racemic mixture. Recent studies in animals as well as humans have established differential pharmacological and toxicological properties of the two enantiomers of PQ. This has been attributed to differential metabolism and pharmacokinetics of individual PQ enantiomers. The aim of the current study is to evaluate the comparative pharmacokinetics (PK), tissue distribution and metabolic profiles of the individual enantiomers in mice.

**Methods:**

Two groups of 21 male Albino ND4 Swiss mice were dosed orally with 45 mg/kg of *S*-(+)-PQ and *R*-(−)PQ respectively. Each of the enantiomers was comprised of a 50:50 mixture of ^12^C- and ^13^C- stable isotope labelled species (at 6 carbons on the benzene ring of the quinoline core). Three mice were euthanized from each group at different time points (at 0, 0.5, 1, 2, 4, 8, 24 h) and blood was collected by terminal cardiac bleed. Liver, spleen, lungs, kidneys and brain were removed, extracted and analysed using UPLC/MS. The metabolites were profiled by tandem mass (MS/MS) fragmentation profile and fragments with ^12^C–^13^C twin peaks. Non-compartmental analysis was performed using the Phoenix WinNonLin PK software module.

**Results:**

The plasma AUC_0-last_ (µg h/mL) (1.6 vs. 0.6), T_1/2_ (h) (1.9 vs. 0.45), and T_max_ (h) (1 vs. 0.5) were greater for SPQ as compared to RPQ. Generally, the concentration of SPQ was higher in all tissues. At T_max_, (0.5–1 h in all tissues), the level of SPQ was 3 times that of RPQ in the liver. Measured C_max_ of SPQ and RPQ in the liver were about 100 and 40 times the C_max_ values in plasma, respectively. Similar observations were recorded in other tissues where the concentration of SPQ was higher compared to RPQ (2× in the spleen, 6× in the kidneys, and 49× in the lungs) than in the plasma. CPQ, the major metabolite, was preferentially generated from RPQ, with higher levels in all tissues (> 10× in the liver, and 3.5× in the plasma) than from SPQ. The PQ-*o*-quinone was preferentially formed from the SPQ (> 4× compared to RPQ), with higher concentrations in the liver.

**Conclusion:**

These studies show that in mice, PQ enantiomers are differentially biodistributed and metabolized, which may contribute to differential pharmacologic and toxicity profiles of PQ enantiomers. The findings on higher levels of PQ-*o*-quinone in liver and RBCs compared to plasma and preferential generation of this metabolite from SPQ are consistent with the higher anti-malarial efficacy of SPQ observed in the mouse causal prophylaxis test, and higher haemolytic toxicity in the humanized mouse model of G6PD deficiency. Potential relevance of these findings to clinical use of racemic PQ and other 8-aminoquinolines vis-à-vis need for further clinical evaluation of individual enantiomers are discussed.

## Background

Primaquine (PQ), the prototype 8-aminoquinoline, has been in clinical use as an anti-malarial drug for over six decades. It is primarily prescribed for radical cure of *Plasmodium vivax* malaria [1, 2], which is still a threat in several regions of the world [3–6]. Interest in this broad-spectrum anti-malarial drug has spiked in recent years with the appreciation that it is a prophylactic agent against all forms of malaria that also has activity against the mature and infective gametocytes of *Plasmodium falciparum* [7–9]. These observations and the recommendations of global health agencies for single-dose PQ use in malaria endemic regions [10–12] raise hope for the utility of PQ in malarial eradication campaigns. However, widespread use of PQ has been limited by the well documented haemolytic toxicity in patients who are genetically deficient in glucose-6-phosphate dehydrogenase (G6PD) activity [13–15]. The recently approved tafenoquine, a long acting 8-aminoquinoline, brought a distinct advantage over PQ in terms of dose regimen (allowing a single dose treatment), but tafenoquine still carries the liability in G6PD deficiency [16–18]. The need for a safer alternative to PQ has driven efforts to better understand of the mechanisms of PQ efficacy and toxicity with a view to improving its therapeutic index.

Although the precise biomolecular mechanism of the anti-malarial activity of PQ is still not fully understood, recent studies have suggested that cytochrome P450-dependent metabolism is necessary for the generation of both the active and the toxic metabolites [19]. Subsequent reports in animal models and clinical studies indicate a role for CYP2D6 in the efficacy of PQ [20–23]. For example, while PQ completely protected wild-type mice from *Plasmodium berghei* sporozoite-induced infection, no protection was observed in CYP 2D knockout mice [24]. Similarly, in a clinical study of a vaccine under development, a strong association was established between CYP2D6 activity and the anti-malarial activity of PQ, with the subjects who are CYP2D6 extensive-metabolizers showing no relapse compared to varying relapses in the intermediate- and poor-metabolizer groups [25]. Initial observations have suggested the role of CYP2D6-mediated formation of an *o*-quinone metabolite of PQ [23–27]. It is not as well established, however, whether the CYP2D6-derived metabolite(s) are also responsible for haemolytic toxicity. Thus far, no direct evidence exists that the metabolites produced in the CYP2D6-rich hepatocytes are transported to erythrocytes through plasma.

Currently available formulations of PQ are a racemic mixture of (−)-(*R*)-PQ (RPQ) and (+)-(*S*)-PQ (SPQ) enantiomers in equal proportions. The metabolism of PQ is enantioselective, as observed in multiple exploratory biotransformation studies [28–32]. Extensive metabolite phenotyping studies have shown that PQ is metabolized via two primary pathways: (a) CYP2D6-dependent pathways of hydroxylation of the quinoline ring; and (b) sequential oxidation of the terminal amine of the side chain by mono-amine oxidase and alcohol dehydrogenase [32, 33]. Direct metabolism of PQ by a phase II conjugation pathway has also been suggested [32]. The RPQ enantiomer is preferentially metabolized to the deaminated products while the SPQ enantiomer yields more of the CYP2D6-dependent oxidative metabolites. With the now known metabolism-dependent activity of primaquine, it is plausible to expect variation in the efficacy and toxicity of the two PQ enantiomers, as has been suggested by some studies. SPQ has not only shown better causal prophylactic and blood schizonticidal efficacy in *P. berghei-*infected mouse malaria models, but also a stronger propensity to cause haemototoxicity in the non-obese diabetic/severe combined immunodeficiency (NOD/SCID) mouse model engrafted with G6PD-deficient human red blood cells, as well as in beagle dogs [34]. Importantly, in Rhesus monkeys both enantiomers have demonstrated essentially the same radical curative activity against *Plasmodium cynomolgi*, but the toxicity profiles were divergent [35, 36]. This suggests that although the metabolic pathways of the enantiomers differ quantitatively, both produce the active/toxic metabolites, albeit to varying degrees.

It is proposed that a better understanding of differences in the pharmacokinetic and pharmacodynamics properties of individual enantiomers of PQ will provide a better understanding of their differential elimination and of the formation and tissue-distribution of their metabolites. This may, in turn, offer insights into the safer use of PQ and other racemic 8-aminoquinolines.

As an anti-infective agent, the concentration of PQ in the target/infected organs, along with its bioactivation, will be significant determinants of effectiveness. It is noteworthy that in mice, PQ has much better activity against the liver stages of the malaria parasite than against mature blood stages. Differential biodistribution of the PQ enantiomers and metabolites to these sites and organs may offer insights into the safety and efficacy of one enantiomer over the other. Thus, besides the differential formation of the active/toxic metabolites, the biodistribution of PQ and its metabolites are considered important for efficacy and toxicity. Clinical studies in progress in parallel [37, 38] will have limited ability to determine relative bio-distribution of individual PQ enantiomers and key metabolites in target organs.

Therefore, the aim of the current study was to conduct a differential pharmacokinetics, metabolism, and tissue biodistribution studies of SPQ and RPQ in uninfected mice.

## Methods

### Primaquine dosing and administration

#### Preparation of ^13^C-labelled primaquine and primaquine enantiomers

Labelling of drug candidates with stable isotopes have afforded convenient tools for tracking drug-derived metabolites in complex matrices by tandem MS/MS profiling of the metabolites [39]. This allows filtering for masses of drug metabolites and resulting fragments with twin peaks attributable to the label. ^13^C-Primaquine, labeled at 6 benzene carbons of the quinoline ring, was synthesized as earlier reported [40]; individual enantiomers (+)-(*S*)-PQ (SPQ) and (−)-(*R*)-PQ (RPQ) were resolved as previously described [34]. Drugs were prepared as the diphosphate salts. Mixtures (1:1) of ^13^C and ^12^C versions of each enantiomer were prepared as previously described [41]. The mixture was diluted in phosphate buffered saline (pH 7.4) for use in in vivo dosing for the tissue distribution studies.

#### Animals

All animal procedures were approved by the Institutional Animal Care and Use Committee of the University of Mississippi. The study was carried out on male Albino ND4 Swiss mice (25–30 g). Forty-five male mice weighing 25 to 30 g were randomly assigned to different groups (3 mice in each group) for the study. In all studies, animals were maintained in 12-h light 12-h dark cycles, had access to rodent chow and water ad libitum, and housed according to the guidelines of the Institute of Laboratory Animal Research (ILAR) Guide (1996) and the SEQUUS Institutional Animal Care and Use Committee [42]. The animals were observed daily for general well-being throughout the study.

#### Administration and sampling

On the day of dosing, 21 mice were orally administered 45 mg/kg SPQ while another 21 mice received RPQ in saline (doses are expressed as PQ base). The remaining 3 mice received the control saline solution. Doses were based on individual animal’s body weight. At each time point (at 0, 0.5, 1, 2, 4, 8, 24 h) randomly selected three mice were euthanized by inhalation of isoflurane anesthetic and blood was collected by direct cardiac bleed into heparinized tubes and centrifuged to separate plasma and erythrocytes pellet. Liver, spleen, lungs, kidneys, and brain were removed, washed extensively with chilled saline to remove adherent blood/tissues, and weighed prior to freezing and storage at − 80 °C until analysis.

### Plasma, tissue and urine analysis

#### Sample preparation

Samples for standard curves were prepared by spiking 200 μL of mice plasma with various concentrations of SPQ and RPQ from 100 to 10,000 ng/mL along with a constant concentration of the internal standard (6-D3-methoxyprimaquine). Tissue samples were homogenized in chilled deionized water using Omni tissue homogenizer. Plasma and homogenized tissue samples were extracted with 2-parts of chilled methanol. The mixtures were vortexed, left on ice for at least 60 min and centrifuged (5000×*g* for 10 min). The separated supernatants were evaporated to dryness using a SpeedVac. The residues were reconstituted in 200 μL methanol, centrifuged and the supernatants were transferred to amber autosampler vials for subsequent injection into the HPLC/MS system. The urinary bladders of three mice from each group were emptied and the collected urine samples were pooled for each group. The urine samples were freeze-dried and reconstituted in methanol as above for HPLC/MS analysis.

#### UPLC/MS instrumentation and analysis

A liquid chromatography–mass spectrometry (LC–MS) method for simultaneous analysis of PQ and its metabolites developed previously was employed in this study [40]. The separation of analytes was achieved within a 25-min run time on a Waters ACQUITY UPLC™ BEH Shield RP18 column (100 mm × 2.1 mm I.D., 1.7 mm) equipped with an LC-18 guard column (Vanguard 2.1 × 5 mm, Waters Corp, Milford, MA, USA) using an ACQUITY UPLC system (Waters Corp, Milford, MA, USA). The UPLC system includes binary solvent manager, sampler manager, column compartment, and photodiode array (PDA) detector. The mobile phase, consisting of water with 0.05% formic acid (A) and acetonitrile with 0.05% formic acid (B), was applied at a flow rate of 0.3 mL/min in the following gradient elution: 0–5 min, 1% B; 5–14 min, 1% B to 13% B; 14–18 min, 13% B to 25% B; 18–22 min, 25% B to 33% B; 22–23 min, 33% B to 43% B, and 23–25 min, 43% B to 100% B. Each run was followed by a 3-min wash with 100% B and an equilibration period of 3.5 min with 99% A/1% B. Column and sample temperatures were set at 50 and 15 °C, respectively. Injection volume was 10 µL, and the strong needle wash (90/10; acetonitrile/water, v/v) and weak needle wash solution (10/90; acetonitrile/water) were used. Peaks were assigned with respect to the mass spectra and retention time of reference compounds or tentatively identified on the basis of high-resolution accurate mass.

Mass spectrometric analyses were performed using electrospray ionization (ESI) in positive mode on a Waters Xevo G2-S QToF mass spectrometer (Waters Corporation, Manchester, UK). The MS instrument was operated in the following conditions: mass scan range of 50–1200 Da, capillary of 3.0 kV, cone of 30 V, source temperature of 80 °C, desolvation temperature of 450 °C, desolvation gas flow of 800 L/h, cone gas flow of 50 L/h, and a collision energy of 6 eV. Leucine-enkephalin was used for the lock mass at a concentration of 5 ng/mL and flow rate of 10 µL/min. Ions [M+H]^+^ (*m/z* 556.2771 Da) and fragment ion (*m/z* 278.1141 Da) of leucine-enkephalin were applied to ensure mass accuracy during the MS analysis. The lock spray interval was set at 30 s, and the data were averaged over three scans. The mass spectrometer was programmed to step between low (10 V) and elevated (10–45 V) collision energies on the gas cell, using a scan time of 0.5 s per function.

Metabolites in the accurate mass data were phenotyped using the Metabolynx® software. The data were searched using predicted metabolite mass, mass defects, isotope, and fragmentation patterns. Each sample was subjected to data acquisition in full scan and data-dependent positive MS/MS, targeted MS/MS (ESI positive ionization mode) and high-resolution MS (HRMS) modes using the Waters ACQUITYTM XEVO QTOF Mass Spectrometer (Waters Corporation, Manchester, UK) connected to the UPLC system via an electrospray ionization (ESI) interface. UHPLC retention time, twin mass peaks with difference of 6 (originating from (13)C(6)-PQ/PQ), and MS–MS fragmentation pattern were used for phenotyping the metabolites [40]. Identification of each metabolite was assisted by its HRMS data, which were used to calculate their elemental compositions. The full scan mass data were screened and filtered using Waters MetaboLynx XS software. The qualitative metabolite identification was performed using this software package. Quantitative metabolite analysis of known metabolites (carboxyprimaquine and primaquine *o*-quinone) was performed using standard calibrations.

#### Pharmacokinetic and statistical analyses

Three mice were sampled for each time point. The data were analysed using the Phoenix WinNonLin (Pharsight Co., Mountain View, CA). Within-time-point data were within 10% of the mean. Pharmacokinetic parameters including the total primaquine systemic exposure (AUC), elimination rate constant (*k*), maximum plasma concentration (*C*_*max*_), time for *C*_*max*_ (*T*_*max*_) and half-life values of primaquine were determined.

## Results

Two sets of 21 mice were dosed with SPQ and RPQ, respectively, with each mouse receiving 45 mg/kg of PQ base. The average body weight of the mice was 34 g while the average PQ dose was 1.53 mg/mouse. Tissue samples (plasma, liver, spleen, lungs, kidneys, and brain) were collected, and primaquine/metabolite concentrations were monitored and profiled against time.

### PQ enantiomer pharmacokinetics and tissue distribution

#### PQ enantiomer plasma pharmacokinetics

**Fig. 1:**
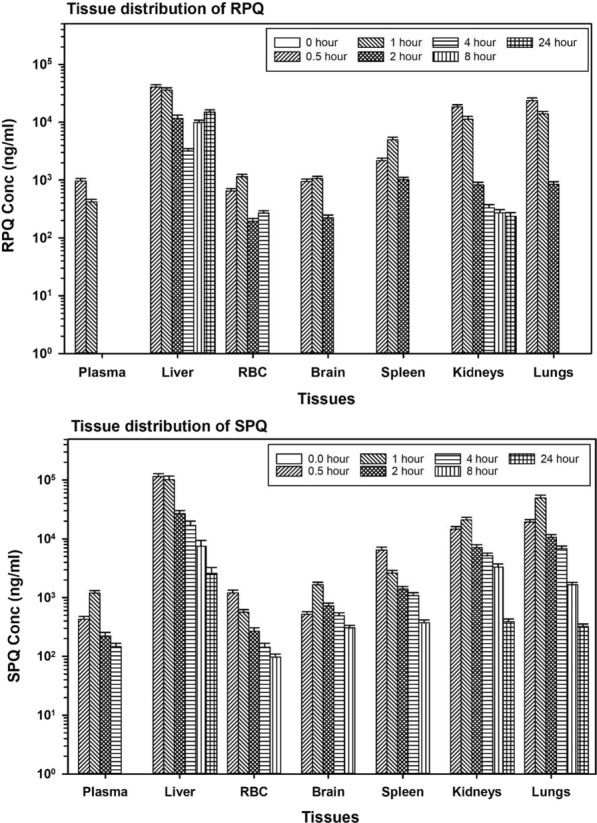
Distribution and time course of primaquine enantiomers in mouse tissues. Data are the average of 3 mice per time point and are presented as mean ± SEM

The basic pharmacokinetic parameters for the two enantiomers are presented in Table 1 show more rapid plasma clearance for RPQ compared to SPQ, with no RPQ detected after 2 h, compared to 8 h for SPQ. Plasma *C*_*max*_ is moderately lower for RPQ, but the *T*_*1/2*_ and AUC_0-last_ are much lower. Peak plasma concentration is achieved more quickly, with *T*_*max*_ at 30 min or earlier. As suggested by the liver concentration–time profile (Fig. 1), it appears that the difference in the *T*_*max*_ of the individual PQ enantiomers may be due to differential tissues perfusion rates, especially from the liver to the blood after the initial hepatic concentration.

**Table 1:** The plasma pharmacokinetic parameters of SPQ and RPQ in mice

	SPQ	RPQ
Lag time (h)	0	0
*C*_*max*_ (µg/mL)	1.05 ± 0.15	0.72 ± 0.11
*T*_*max*_ (h)	1	0.5
*T*_*last*_ (h)	8	2
AUC_0-last_ (µg h/mL)	1.58	0.64
*T*_*1/2*_ (h)	1.9	0.45

*C*_*max*_ maximum plasma concentration, *T*_*max*_ time for *C*_*max*_, *T*_*last*_ time for last measurable plasma concentration, *AUC* area under the plasma concentration–time curve, *T*_*1/2*_ half-life

#### PQ enantiomers tissue distribution

Primaquine is preferentially concentrated in the liver compared to plasma and most other organs (Fig. 1), especially with SPQ. The concentrations of the SPQ enantiomer were higher than RPQ in the liver tissues at all the time points. At *T*_*max*_, the concentration of SPQ in liver is about 3 times that of RPQ; (115 vs. 40 mg/mL). At this point, the liver alone accounts for 20% of administered SPQ. Since the first sampling point of 30 min also happened to be the *T*_*max*_, the actual *T*_*max*_ for RPQ might be earlier than 30 min. This suggests a rapid absorption and distribution of PQ enantiomers to the liver. At the *T*_*max*_, measured C_*max*_ of SPQ and RPQ in the liver was about 100 and 40 times the value in the plasma respectively. In the other organs, the concentration of the SPQ enantiomer is also preferentially higher than in plasma. For example, at *T*_*max*_, the concentration of SPQ is about 5 times higher in the spleen and lungs, and about twice as high in the brain and kidneys, compared to plasma.

There is a notable difference in the time course of the liver profiles of RPQ and SPQ, with a biphasic increase in liver content of RPQ. The second peak of hepatic RPQ occurs between the 4-h and 8-h time points and increases further at 24 h. This biphasic pattern for tissue distribution is absent with SPQ.

### Major metabolites

#### Plasma and tissue concentrations of carboxyprimaquine

**Fig. 2:**
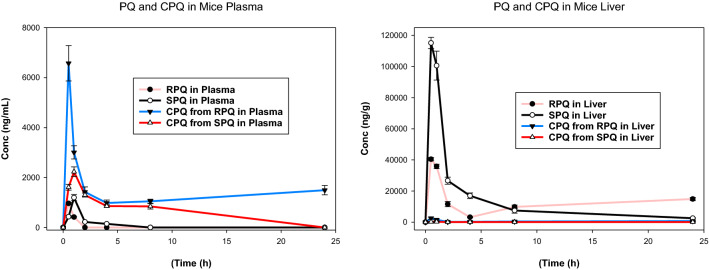
Time course of concentrations of primaquine and carboxyprimaquine in plasma and liver after RPQ and SPQ administration. Data shown are mean ± SEM (n = 3) in mouse plasma (left panel) and liver (right panel)

**Fig. 3:**
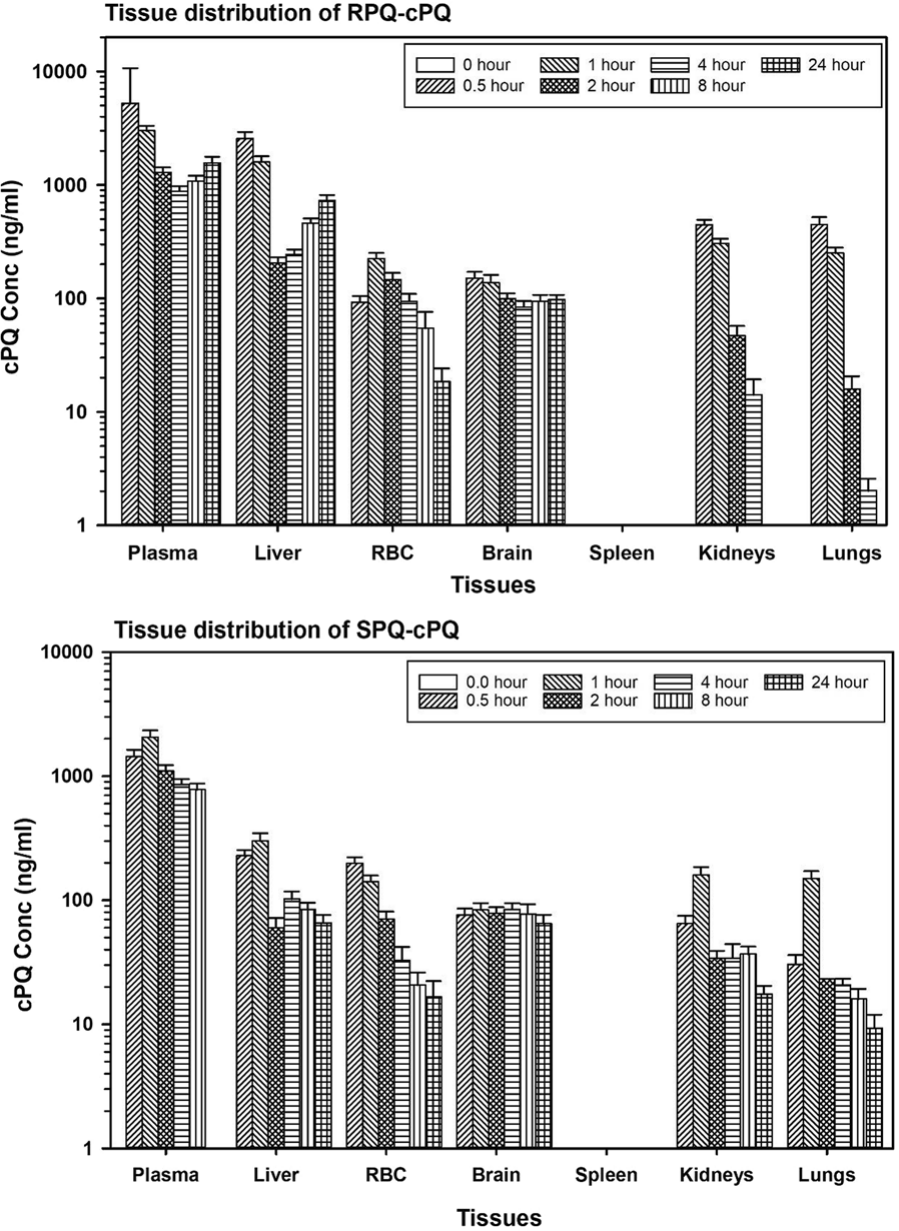
Comparative tissue distribution of carboxyprimaquine formed from primaquine enantiomers. Data are the average ± SEM of 3 mice per time point

Carboxyprimaquine (cPQ), the primary metabolite of primaquine, was the most abundant metabolite formed and was found in plasma and all the tissues. These results are shown in Figs. 2 and 3. Figure 2 compares the relative time-course for formation of cPQ from individual PQ enantiomers, and their comparative profiles in plasma and liver. As expected, based on previous studies, RPQ consistently generated more cPQ as compared SPQ. CPQ was a major metabolite in plasma, with significant amounts found in the liver. The concentrations of cPQ are higher in plasma than in liver, especially for RPQ, even though the parent PQ content of both enantiomers is much higher in liver than in plasma.

The formation of cPQ is faster with RPQ, peaking at 30 min in both plasma and liver (first time-point) compared to 1 h with SPQ. At Tmax, the level of cPQ from RPQ in liver is about 10× greater than the level formed from SPQ. CPQ is about 3.5-fold higher in the plasma after RPQ administration, as compared to SPQ. The biphasic pattern observed with RPQ in liver (Fig. 1) is mirrored in the biphasic appearance of cPQ in plasma from RPQ, but this is absent or minimal with SPQ.

Carboxyprimaquine (cPQ) was found in all the organs except in the spleen (Fig. 3). Except for plasma, cPQ is much lower in the tissues compared to the parent RPQ or SPQ. In plasma, for both enantiomers, the concentration of cPQ far exceeds that of PQ at the T_max_.

### Primaquine 5,6-*o*-quinone

**Fig. 4:**
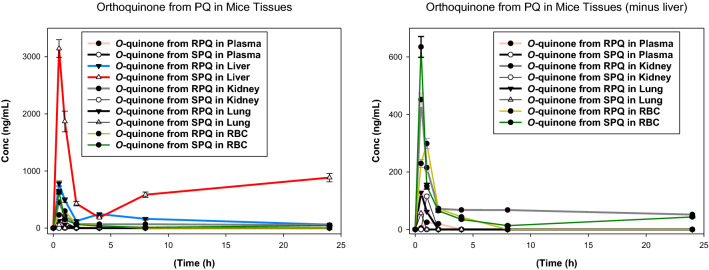
Time course of concentrations of PQ-5,6-*o*-quinone after administration of individual RPQ and SPQ. Data shown are mean ± SEM (n = 3) in mouse plasma, liver (left panel) or plasma and erythrocytes (right panel)

**Fig. 5:**
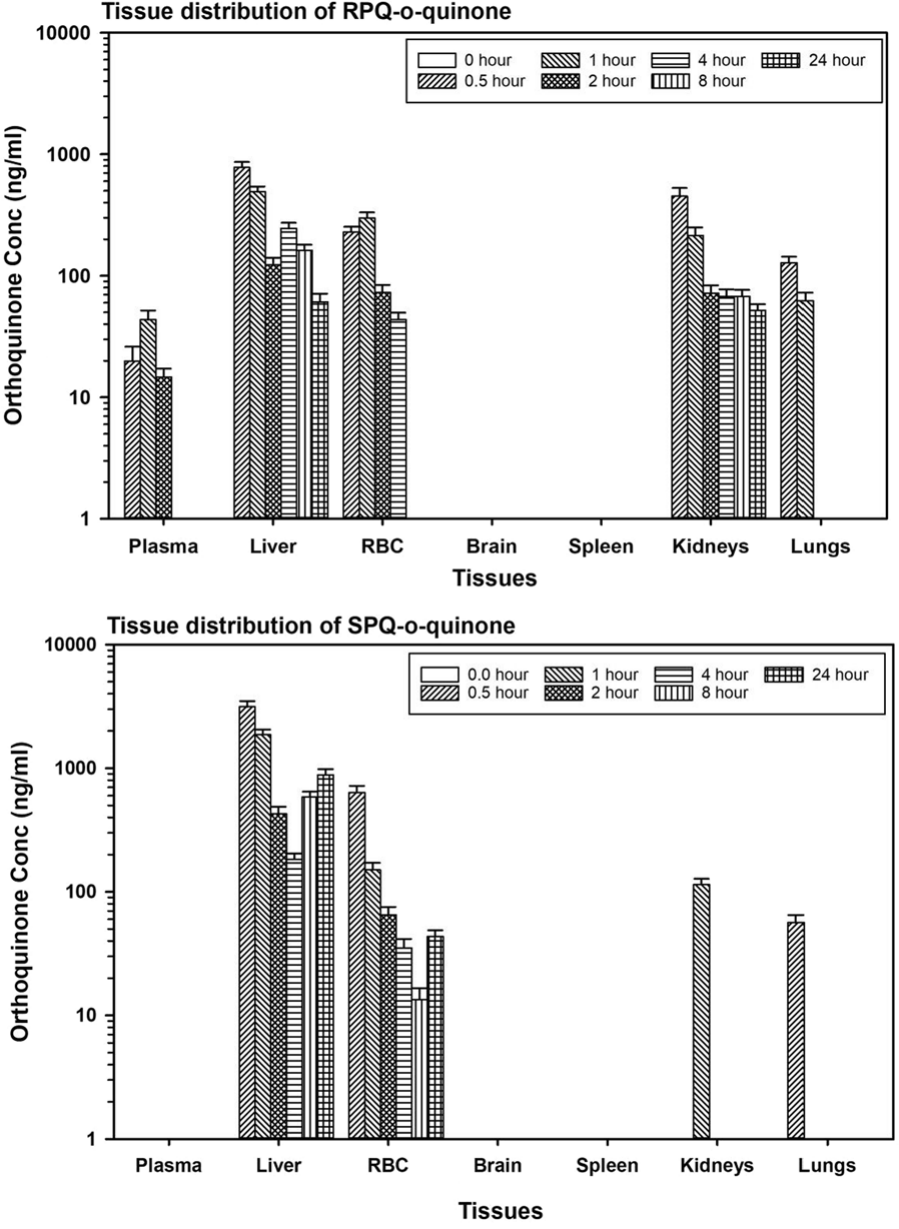
Time course of concentrations of PQ-*o*-quinone after RPQ and SPQ administration. Data are the average ± SEM of 3 mice per time point

Primaquine 5,6-*o*-quinone is a stable surrogate of the reactive, unstable 5-hydroxyprimaquine metabolite. Figures 4 and 5 compare the kinetics of primaquine *o*-quinone generated from individual PQ enantiomers. *O*-quinone was primarily formed in the liver, with about 4 times more from SPQ than from RPQ. The *o*-quinone was also observed at appreciable concentrations in RBCs. In plasma, only trace amounts of *o*-quinone were detected from RPQ, and none from SPQ. The concentration of *o*-quinone formed from SPQ in RBC is about twofold higher than formed from RPQ at the T_*max*_, thus mirroring the plasma PQ profiles, rather than the plasma *o*-quinone profiles.

### Other plasma and tissue metabolites

A number of other PQ metabolites were identified in plasma and tissues. The identity of these metabolites was established with the MetaboLynx™ XS application manager (Waters USA) based on retention time, accurate mass and high-resolution mass fragmentation pattern with twin stable isotope peaks [40]. The summary of these metabolites (with their putative identity) is provided in Table 2. Secondary metabolites (Phase II metabolism) found in this mouse tissue study include the *N*-glucuronide of primaquine (m/z 436) and phenolic glucuronides of two hydroxy-primaquines (m/z 452), along with a glycosylated primaquine derivative (m/z 422).

**Table 2:** Other metabolites formed by PQ enantiomers as observed in different mouse tissues

Mass (M + 1)	Retention time (min)	Putative identity	Key findings
274.1544	2.35	Hydroxylation and quinone-imine formation	Formed from both enantiomers, found only in the plasma
332.1604	7.7	Acetylated dihydroxy-PQ quinone-imine	Formed from RPQ and found in plasma and liver
422.1929	4.9	Glycosylated primaquine	Higher amount formed from SPQ than RPQ; found in the plasma and liver
436.1992	2.17	Primaquine glucuronide	Present only in the plasma with no remarkable enantiomeric difference in the profiles
452.2027	1.9	Hydroxy-primaquine glucuronide	Formed only from the SPQ enantiomer and found only in the in the plasma
452.2027	9.26	Hydroxy-primaquine glucuronide	Formed from both enantiomers and found in the liver, RBC, kidneys and lungs. No remarkable enantioselectivity
480.1989	7.9	Primaquine-*N*-carbamoyl glucuronide	Formed from both enantiomers; found only in the plasma

An apparent glucuronide of primaquine (m/z 436, RT 2.17 min) was found in the plasma only, with no remarkable differences in the enantiomers. Hydroxy-primaquine glucuronides (m/z 452) were also major secondary metabolites of PQ found in this study. Two different glucuronides (distinguished by their retention times 1.9 and 9.26 min), likely formed from the glucuronidation of two of 2-, 3-, 4- or 5-hydroxyprimaquines, were detected. The first hydroxy-primaquine glucuronide (RT 1.9 min) was found only in the plasma and was exclusively formed from the SPQ enantiomer. The second glucuronide (RT 9.26 min) had a wider distribution across the tissues (liver, RBC, kidneys, and lungs), and was formed from both enantiomers.

A glycosylated PQ (RT 4.9 min) was more abundantly formed in the liver than in the plasma; but was relatively more abundantly generated from SPQ in the liver and from RPQ in the plasma. Other metabolites detected include an apparent acetylated dihydroxy-primaquine quinone-imine (m/z 332) which was formed only from RPQ and found only in the plasma and liver; primaquine-*N*-carbamoyl-glucuronide conjugate (m/z 480) was found only in the plasma and formed from both enantiomers; and there were traces of a PQ quinone-imine (m/z 274), presumably derived from 5-OH-PQ—and an intermediate leading to the PQ-o-quinone—formed from both enantiomers and found only in the plasma.

### Urine metabolites

Several of these metabolites, along with additional ones, were recovered from the mouse urine. Table 3 shows the relative presence of the identified metabolites from the two PQ enantiomers. Shaded rows indicate the metabolites also seen in plasma or tissues.

**Table 3:** Urinary metabolites (and their putative identities) detected in mice following the administration of RPQ and SPQ

Mass (M + 1)	Retention time (min)	Description	Relative presence	
			RPQ	SPQ
246.1585	4.3	Demethylated primaquine	+	+
260.1408	1.87	Primaquine-*o*-quinone	+	+
261.1609	8.7	Oxidative deamination to primaquine alcohol	++	+
274.1544	2.35	Hydroxylation and quinone-imine formation	+	−
276.1690	4.18	2-Hydroxy-primaquine	−	+
276.1691	4.88	3-Hydroxy-primaquine	+	−
289.1531	10.04	CarboxyPQ *o*-quinone	+	−
290.1510	3.45	Dihydroxy-PQ quinone imine	+	−
332.1604	7.7	Acetylated dihydroxy-PQ quinone-imine	++	+
422.1929	2.3	Demethylation + glucuronidation	+	+
422.2266	4.9	Glycosylated primaquine	+	+
452.2027	1.9	Hydroxy-primaquine glucuronide	−	+
452.2027	2.37	Hydroxy-primaquine glucuronide	+	−
452.2027	3.4	Hydroxy-primaquine glucuronide	+	+
452.2027	4.3	Hydroxy-primaquine glucuronide	−	+
467.1642	5.6	Glucuronide of hydroxylated carboxyprimaquine	−	+
467.1642	7.27	Glucuronide of hydroxylated carboxyprimaquine	+	−
480.1989	7.9	Primaquine-*N*-carbamoyl glucuronide	+	+
494.2125	3.0	Primaquine acetylation, hydroxylation and glucuronidation	+	−
494.2125	2.9	Primaquine acetylation, hydroxylation and glucuronidation	−	+
494.2125	4.7	Primaquine acetylation, hydroxylation and glucuronidation	−	+

(−) denotes the absence of the metabolite. Where present in both enantiomers, relative amount is denoted with (+) and (++) denoting low and high amounts respectively

## Discussion

This study presents a comprehensive pharmacokinetic and tissue bio-distribution analysis of PQ and key metabolites in healthy mice administered a single dose of the individual PQ enantiomers. It is well established that for chiral drugs, pharmacological and toxicological effects of the enantiomers may vary substantially; this divergence may be due to pharmacodynamic considerations (e.g., target receptor interactions) but may also be dependent on differential absorption, distribution, metabolism, and excretion profiles [43]. This phenomenon has been shown to be of critical importance for several drugs and antimalarial drug–drug interactions [41, 44].

For PQ, evidences from a number of in vitro [28, 29] and in vivo [31, 44] studies have established that the metabolism to cPQ, which is a major metabolic route, is distinctly stereoselective, with RPQ giving rise to more of the cPQ metabolite, likely because it is a better substrate for monoamine oxidases than SPQ [30]. Differences in in vivo pharmacodynamic and toxicological profiles with the PQ enantiomers have been observed in other studies in mice, dogs [34] and primates [35, 36].

Based on in vitro studies with individual PQ enantiomers, it was established that besides metabolism of PQ to cPQ, other important PQ metabolic pathways also show potential stereoselectivity with regard to the PQ enantiomers. The SPQ enantiomer, more than the RPQ, is susceptible to the quinoline ring oxidation via CYP-dependent metabolism [30]. This finding points toward the likelihood that SPQ may display greater anti-malarial (radical curative) potency than RPQ. However, organ-specific uptake of parent drug and production of the metabolites will also be important considerations for understanding of the pharmacodynamics of PQ, along with the distribution and further metabolism of metabolites.

It is of interest that the half-life of both enantiomers in tissues appears to be much longer than the half-life in plasma, reflecting the known high volume of distribution of this drug [45]. This is consistent with a slow back equilibration of the drug from tissue binding sites before elimination in the liver. However, and most noticeable for the R enantiomer relative to the S analog, the concentrations in the liver at long time intervals are much greater than in other ‘binding’ tissues with the possible exception of the kidney. This implies the presence of non-metabolism related binding sites in the liver. The data from current study also suggests that the elimination of the SPQ from the liver is faster and more consistent with its plasma half-life, as compared with that of the RPQ, again implying something special about the R isomer’s non-metabolic binding in the liver. Of interest, SPQ has been reported to show a higher activity against sporozoite-induced liver stage malaria infections in mice [34]. Whether this is due to relative sequestration of the R isomer and hence lower metabolic activation to the active species is not yet clear, but it raises the possibility that preservation of the R species in the liver may be relevant to activity of PQ towards mature and infective gametocytes, whether with or without further activation. In addition, whether any such ‘activated metabolites’ differ from those that elicit radical cure of *P. vivax*. The extended high levels relative to plasma in the lungs are notable, especially for the SPQ enantiomer. Further studies in this regard may explore potential activity of PQ or other 8-aminoquinolines against pulmonary parasite infections. PQ-based combinations have shown utility for treatment of *Pneumocystis jirovecii* (carinii) pneumonia causing lung cystic lesions [46, 47].

Collectively, the tissue distribution data showing sustained concentrations of both isomers, relative to plasma levels, emphasize that the biological half-life of the drug, towards both the therapeutic and toxic endpoints, is likely to be much greater than that predicted by plasma half-life alone, and may further differ depending on the endpoint, hepatic sporozoites or circulating gametocytes eradication.

Formation of cPQ showed significant enantioselectivity with markedly higher levels of R-cPQ (Fig. 2). As expected from its much smaller Vd compared to that that of PQ [29, 45], it was more concentrated in the plasma than in the tissues. Of note, the concentrations of cPQ for both isomers in plasma and other tissues were maintained for up to 24 h, again raising the issue of the potential role of cPQ in the therapeutic and toxicological responses to PQ in experimental animals and patients [32]. Identification of carboxyPQ *o*-quinone in the urine indicates that ring oxidation of cPQ occurs in vivo and could contribute to the biological activities of PQ. Whether this pathway is quantitatively important for therapeutic/toxicological effects remains to be determined.

As illustrated in Fig. 2, R-cPQ appears to show a biphasic response, an initial rapid decrease, presumably reflecting both metabolic clearance and tissue distribution, followed by an increase starting at about the 2 h time point. The mechanism underlying this increase and persistence is unclear; an enterohepatic circulation of a cPQ metabolite such as the acyl-glucuronide is conceivable especially because the presence of gall bladder in mice favors intermittent dumping of bile followed by reabsorption. In view of the marked difference in the initial clearance of RPQ vs SPQ, it seems reasonable that if the cPQ metabolite plays a role in the formation of the ultimate active/toxic metabolites, its contribution would be most marked for the R isomer. This study provides extended results on low tissue distribution of cPQ and supplement earlier clinical studies on differential PK profiles of cPQ enantiomers [31].

Primaquine 5,6-*o*-quinone, potentially generated through CYP2D6 meditated pathway has been implicated in the therapeutic/toxic actions of PQ [30, 48, 49]. In this study, low concentrations of *o*-quinone of the R isomer were detected in plasma, but none from the S isomer (Fig. 5). The *o*-quinone from both isomers was present in significant concentration in the liver, and to smaller extent, in the kidney and lungs. Earlier clinical studies have also failed to detect primaquine-*o*-quinone in plasma of healthy human volunteers treated with PQ single dose [32, 50], possibly reflecting rapid uptake into RBCs or distribution to tissues. As shown in Fig. 5, in spite of low plasma concentrations, the *o*-quinone was present in substantial amounts (hundreds of ng/ml) in RBCs. There was persistent presence of this potentially haemotoxic species in both the RBCs and liver long after the elimination of the parent compounds. The origin of *o*-quinone in the RBC is of interest. The capacity of oxyhaemoglobin to mediate the oxidation of drugs is long known, albeit sluggish as compared to the hepatic P450 system. With regard to PQ, it has been shown that the *o*-quinone can be produced from PQ in an in vitro incubation with RBC [51]. The contribution of RBC in situ oxidation vs hepatic P450 formation/transport to the RBC remains to be determined.

Enantioselectivity in metabolic clearance of drugs is well known. The enantioselectivity of PQ metabolism is prominent: the long recognized major difference in rate of MAO-induced formation of cPQ for the two enantiomers can be extended to other pathways. The cPQ formation from RPQ results in two species (parent and metabolite) with markedly different tissue distribution and persistence; both have the metabolic potential for activation(s) needed for the therapeutic and haemotoxicity of the parent compound. The hydroxylated metabolites, including PQ-*o*-quinone (presumably CYP-dependent), is observed with both enantiomers; however, the specific metabolites formed and tissue distribution varies between the two enantiomers. Other metabolites with notable enantioselectivity include the glucosyl PQ and hydroxyPQ glucuronide, both of which were preferentially generated from the SPQ. A large number of metabolites were found in the mouse urine with a number of them showing enantioselectivity. Many of these metabolites have been earlier detected in urine in a clinical study [32].

The *o*-quinone in liver tissue also reflects a biphasic profile, especially with the SPQ; this suggests some recycling of this or other (upstream) metabolites. This may be attributed to the difficulty of isolating the unstable intermediates 5-hydroxy-PQ, PQ-quinone-imine, and 5,6-dihydroxy-PQ. If one or more of these has an affinity for the tissues that stabilizes them or delays their release and breakdown to the *o*-quinone, this may also account for this puzzling finding.

The presence of *o*-quinone in the RBCs is remarkable. While it is not clear whether the *o*-quinone produced in the liver is transported to the RBC, an earlier study has shown that *o*-quinone can be produced via direct oxidation of PQ on in vitro incubation with RBCs [51]. The RBC concentration of *o*-quinone however, raises questions regarding its role in the haemotoxicity associated with PQ, and/or the gametocytocidal activity of PQ [10]. A recent report has suggested the role of CYP mediated pathway in gametocytocidal activity but not with treatment safety [52].

This study has further highlighted the potential role of tissue distribution in PQ activity/toxicity, and why this role may not be the same for both enantiomers. Together with earlier findings, the current study suggests that PQ enantiomers may be regarded as two different drugs with remarkable differences in pharmacokinetics, metabolism, and tissue distribution, which translate into different efficacy and safety profiles of individual enantiomers. Ongoing clinical studies with individual PQ enantiomers in normal and G6PD deficient individuals [38, 53] will further inform the applicability of these current findings.

## Conclusion

PQ enantiomers are differentially biodistributed and metabolized in mice, suggesting potential variation in the pharmacologic and toxicity profiles. Both enantiomers are extensively metabolized, but the specific metabolite patterns vary substantially. Systemic exposure to parent SPQ is far greater than observed with RPQ, and the conversion of RPQ to carboxy-primaquine is much higher than for SPQ. The findings are consistent with the higher activity of SPQ observed in the mouse causal prophylaxis test, and higher toxicity in the mouse model of haemolytic toxicity in G6PD deficiency [34]. The relevance of these findings to clinical use of racemic PQ and other 8-aminoquinolines vis-à-vis the actions of individual enantiomers remains to be explored in clinical studies.

## Data Availability

All data generated or analysed during this study are included in this published article and its Additional files.
